# Amylin vasculopathy impairs cerebral Aβ efflux through altering cerebral vasodilation

**DOI:** 10.1002/alz.093349

**Published:** 2025-01-03

**Authors:** Ravichandra S Davargaon, Noah S Leibold, Nirmal Verma, David K Powell, Florin Despa

**Affiliations:** ^1^ University of Kentucky, Lexington, KY USA

## Abstract

**Background:**

Impaired interstitial fluid drainage in the brain is indicated by the presence of perivascular β‐amyloid (Aβ) deposits and is attributed to alterations in contractility and relaxation of vascular smooth muscle cells (SMCs). The brain microvasculature in Alzheimer disease (AD) accumulates amyloid‐forming amylin secreted from the pancreas. Here, we tested the hypothesis that cerebrovascular amylin deposits perturbs cerebral Aβ efflux by impairing cerebral vasodilation.

**Methods:**

Using transgenic rats expressing amyloid‐forming human amylin in the pancreas (HIP rats) (aged 16‐months) and wild‐type (WT) littermates that express non‐amyloidogenic rat amylin, we conducted comparative analyses of cerebral blood flow (CBF), pressure myography in isolated pial arteries and vascular SMC oxidative stress experiments.

**Results:**

Longitudinal brain MRI measurements revealed consistent structural alterations that progressed more rapidly with aging in HIP vs. WT rats, leading to 14.9% reduction in CBF in HIP rats. Plasma nitrite and nitrate, stable nitric oxide (NO) end products, were increased in HIP vs. WT by 84.7% and 24.87%, respectively. Pressure myography experiments using pial arteries showed that both WT and HIP arteries developed arterial tone (e.g. pressure‐induced constriction); however, arteries from HIP rats show significant elevations (56.9‐142.3%) in arterial tone compared to WT rats at physiologically‐relevant intravascular pressures (e.g. 60‐100 mmHg). Consistent with these results, vascular SMCs from HIP rats showed elevated (12.6% increase) lipid peroxidation, which was replicated in SMCs incubated with exogenous human amylin (29.6%). Increased lipid peroxidation contributes to oxidative stress in the vascular wall and reduces NO bioavailability, altering vasodilatory function. Both arginase activity and expression (of Arginase 1 and 2) were increased in brain microvascular lysates from HIP rats compared to those from WT by 17.6%, 63.9%, and 57.8%, respectively, suggesting arginase‐NO dysregulation. A possible impact of increased blood amylin concentration on cerebrovascular arginase‐NO regulation was further tested in brain microvascular lysates from rats intravenously injected with amyloid‐forming human amylin (55.0% reduction in arginase activity).

**Conclusion:**

Our results indicate perivascular Aβ deposits in the setting of AD are potentially linked to amylin vasculopathy and altered spontaneous contraction/relaxation of cerebrovascular SMCs. Future experiments will focus on delineating molecular markers of amylin‐induced alterations of SMC contractile phenotype.

